# Outcomes With Early Cardiac Catheterization in Out of Hospital Cardiac Arrest Survivors and Utility of a Prognostic Scoring System

**DOI:** 10.7759/cureus.16775

**Published:** 2021-07-31

**Authors:** Deepak Vedamurthy, Shilpa Singh, Keshab Subedi, Kirk N Garratt, Neil J Wimmer

**Affiliations:** 1 Hospital Medicine, Christiana Care Health System, Newark, USA; 2 Internal Medicine, Christiana Care Health System, Newark, USA; 3 Biostatistics, Christiana Care Health System, Newark, USA; 4 Cardiology, Christiana Care Health System, Newark, USA

**Keywords:** neurologic prognosis, out of hospital cardiac arrest, coronary angiography, 30 day mortality, cardiac arrest hospital prognosis score

## Abstract

Objectives

A retrospective study in patients presenting out of hospital cardiac arrest (OHCA) to assess the impact of early cardiac catheterization on survival and cerebral performance category (CPC) on discharge.

Background

The role of early coronary angiography in OHCA patients remains controversial. The cardiac arrest hospital prognosis (CAHP) scoring system has not been validated in the US population.

Methods

Inclusion criteria were OHCA patients with a sustained return of spontaneous circulation (ROSC), presumed cardiac cause of arrest, and elements to calculate CAHP score. We compared in-hospital mortality rates and final inpatient CPC in patients who underwent early cardiac catheterization to those with delayed or no cardiac catheterization. We assessed the performance of the CAHP score in the entire OHCA population using receiver-operator curve (ROC) analysis.

Results

A hundred and fifty-eight patients were included, of which 39 underwent early cardiac catheterization. The mortality rate of the early catheterization group was lower than the delayed or no catheterization group (41% vs 61.3%, p=0.02); the Early cardiac catheterization group had more favorable final hospital CPC scores overall (53.8% vs 24.3%, p<0.001). However, when risk-adjusted, there was no benefit in early catheterization for mortality or CPC level in any of the CAHP score subgroups. CAHP scores showed good discrimination with c-statistics of 0.85 for mortality and 0.90 for the CPC category.

Conclusion

Early use of cardiac catheterization in OHCA patients with sustained ROSC was not associated with lower mortality rates or higher rates of favorable neurologic recovery when adjusted for baseline risk factors in each of the different CAHP score-based sub-groups. This was despite a higher proportion of patients with STEMI in the early catheterization group. We demonstrated a good fit between observed outcomes and outcomes predicted by the CAHP scoring system.

## Introduction

Out-of-Hospital Cardiac arrest (OHCA) is a significant worldwide public health problem affecting approximately 395,000 per year and the overall survival is very low (estimated to range from 2 to 10%) [1]. Cardiac arrest could be due to acute coronary syndrome or other cardiac or non-cardiac etiologies. Multi-society guidelines from 2019 recommend early coronary angiography and re-perfusion for all post-arrest patients manifesting with STEMI after the return of spontaneous circulation (ROSC) is achieved and prompts consideration for cardiac catheterization in most patients when a cardiac cause of arrest is suspected, even if there is no ST-segment elevation on a post-resuscitation ECG [2]. Recent literature debates the role of coronary angiography in patients, especially those with a high likelihood of death [3]. The most recent Society for Cardiovascular Angiography and Intervention (SCAI) consensus statement notes that among comatose OHCA patients with ROSC and STEMI, there are no randomized controlled trials to support favorable neurological outcomes or survival benefit of immediate angiography [4].

In order to prognosticate outcomes in OHCA patients, the cardiac arrest hospital prognosis (CAHP) scoring system was developed. The CAHP score was developed based on a French national registry. This risk tool was generated from an all-comer patient population following cardiac arrest without regard to the finding of ST-segment elevation on the ECG. The dataset utilized relevant system, demographic, and clinical data points such as time from collapse to basic life support, duration of cardiopulmonary resuscitation (CPR), age, arterial pH upon arrival, and others. to predict neurological outcomes after arrest and demonstrated a good fit between high-risk scores and poor prognostic outcomes [5].

There is little compelling data that performing early cardiac catheterization in patients with high CAHP scores after OHCA improves outcomes, irrespective of the presence of ST-elevation myocardial infarction (STEMI) [6]. Coronary angiography may subject the patient to risks (vessel injury, life-threatening cardiac arrhythmias, death, renal injury, blood loss) without offering any survival benefit, especially in those patients who are suspected to have severe hypoxic-ischemic encephalopathy after the cardiac arrest. It would be helpful to determine if performing an early coronary angiogram (CAG) in patients with high CAHP scores (which indicates poor prognosis) after OHCA impacts outcomes. 

We undertook this study to assess the relationship between early cardiac catheterization and survival and final hospital cerebral performance category scores among OHCA patients with ROSC; patients were studied as an aggregate group and after being stratified into risk categories according to CAHP scores. We hypothesize that deferring urgent cardiac catheterization in OHCA patients with high CAHP scores (irrespective of the presence of STEMI) will lead to similar outcomes as performing early catheterization. We also tested the performance of the CAHP score in predicting mortality and cerebral performance score in our patient population.

## Materials and methods

We retrospectively analyzed data from our cardiac catheterization database and electronic health records to electronically identify all patients at ChristianaCare Health System (CCHS) who presented to the hospital with OHCA between January 1, 2016, and October 31, 2019. CCHS is the largest tertiary referral center in the state of Delaware with a county-wide catchment area covering approximately 2/3 of the state’s population. The electronic charts of the patients meeting inclusion criteria were then manually reviewed for exclusion criteria and clinical outcomes. Patients were included if they survived for at least one hour in the hospital after presenting with OHCA, which was accepted as a surrogate for durable ROSC. Patients with obvious non-cardiac causes of arrest like trauma, motor vehicle accident, asphyxiation, pulmonary embolism, or chronic lung disease exacerbation were excluded. Patients were included irrespective of the underlying cardiac rhythm at the time of arrest (ventricular fibrillation/ pulseless ventricular tachycardia/ pulseless electrical activity/asystole). Patients for whom incomplete documentation regarding the resuscitation prevented calculation of a CAHP score were excluded. This study was approved by the CCHS Institutional Review Board.

Operational definitions

Patients were defined as having early catheterization if they were taken to the catheterization lab within 24 hours of presentation to the emergency room. Patients who underwent cardiac catheterization at any time during the hospital stay beyond 24 hours were categorized as having delayed catheterization. Patients who did not have any catheterization were categorized as having no cardiac catheterization. Since the primary aim was to identify any relationship that may exist between early cardiac catheterization and survival and neurologic recovery, patients with delayed and with no cardiac catheterization were grouped together.

Patient comorbidity was quantified using the Charlson comorbidity index [5]. Neurological status was assessed by chart review using the five-level cerebral performance category (CPC) scale, with a CPC level of 1 (good recovery) or 2 (moderate disability) classified as favorable neurological status, and a CPC level of 3 (severe disability), 4 (vegetative state) or 5 (death) classified as unfavorable neurological status [5].

The CAHP score is a simple and objective score based on admission parameters that permit the prediction of neurological outcomes in patients admitted to the hospital following OHCA. It includes seven variables associated with poor prognosis (age, non-shockable rhythm, time from collapse to onset of basic life support (BLS), time from BLS to ROSC, location of cardiac arrest (home versus public setting), epinephrine dose used during resuscitation, and arterial pH upon hospital admission) and has a high discrimination value [5]. CAHP scoring system considers 0-150 to reflect low risk, 151-200 to reflect medium risk, and >200 to reflect high risk of death.

Analysis and outcomes

The primary endpoint of this study was unadjusted in-hospital mortality. A secondary endpoint was an unadjusted cerebral performance score at the time of discharge. Risk-adjusted endpoint analyses using age, gender, Charlson comorbidity scores, the timing of cardiac catheterization, and CAHP score at baseline were also calculated. 

Data sources 

Data were extracted manually by two of the authors (DV & SS) over a three-month period from ChristianaCare’s electronic health record environment (Powerchart™, Cerner, North Kansas City, MO). All ChristianaCare Emergency Department and inpatient clinical information have been captured in this electronic health record since 2005. 

Statistics

The baseline clinical and demographic characteristics of the study population were summarized using count and percentage for categorical variables, the mean and standard deviation for continuous variables with normal distribution, and median and interquartile range (IQR) for continuous variables with skewed distribution. We compared the baseline clinical and demographic characteristics between patients with early catheterization and patients with delayed or no catheterization using the chi-squared test, t-test, and Wilcoxon-rank sum test. The outcomes of interest (mortality and cerebral performance score) were evaluated between patients with early catheterization and patients with delayed or no catheterization using the chi-squared test in the overall study population and in the subgroup of patients with low, medium, and high CAHP scores. Multivariable logistic regression models of mortality and cerebral performance score (favorable vs unfavorable) were evaluated using age, sex, Charlson comorbidity score, catheterization time, and CAHP score groups as covariates. An interaction term between catheterization time and CAHP score group was introduced in the model to explore if the effect of catheterization time was modified by CAHP score.

The validity of CAHP score in predicting mortality and final pre-discharge cerebral performance score was evaluated using receiver-operator curve (ROC) analysis. ROC curves associated with logistic regression-based predictions of mortality and cerebral performance scores as a function of CAHP score were plotted. Model discrimination was evaluated using the area under curve (AUC) parameter. All the statistical analyses were performed using SAS 9.4®.

## Results

Study population 

We identified 1021 patients who met the criteria for OHCA between January 1, 2016, and October 1, 2019. We excluded 401 patients who had a hospital length of stay of less than one hour as these patients were presumed to have failed to achieve durable ROSC. Of the remaining 620 patients, 453 were excluded because they had an obvious non-cardiac cause of arrest or we found insufficient documentation of elements required to calculate a CAHP score. The remaining 158 patients form the study population. 

Clinical and procedural characteristics 

The baseline clinical characteristics of the study population are summarized in Table 1. A mean age of 66 years was observed, 72% were male, 69% were non-Hispanic whites, and 20% presented with electrocardiographic (ECG) findings consistent with STEMI. Thirty-nine (24.7%) patients underwent early catheterization, 22 (13.9%) patients underwent delayed cardiac catheterization and 97 (61.4%) patients underwent no catheterization. ECG findings consistent with STEMI were present in 25 (64%) patients with early catheterization and 7 (5.8%) patients with delayed or no catheterization. The median Charlson comorbidity index in the entire population was 2.0; the delayed/no catheterization group had a median score of 3.0, while the early catheterization group had a median score of 0.0. Univariate comparisons demonstrated that compared with those with delayed or no cardiac catheterization, patients undergoing early catheterization were more likely to have ECG findings indicating STEMI (p = 0.01), younger (p = 0.01), and less likely to have congestive heart failure, diabetes, renal insufficiency or high CAHP scores (all p values < 0.05). 

**Table 1:** Baseline Clinical Characteristics Characteristics of the study population grouped by timing of cardiac catheterization. The numbers are count and percentage unless otherwise noted.

Variable	All (n= 158)	Delayed/None Catheterization (n=119)	Early Catheterization (n=39)	P-value
Age (mean, SD)	66.31 (13.41)	67.90 (13.68)	61.45 (11.41)	0.005
Sex				0.055
Female	43 (27.22%)	37 (31.09%)	6 (15.38%)	
Male	115 (72.78%)	82 (68.91%)	33 (84.62%)	
Race				0.062
Black	32 (20.25%)	29 (24.37%)	3 (7.69%)	
White	109 (68.99%)	79 (66.39%)	30 (76.92%)	
Other	17 (10.76%)		17 (12.50%)	
Ethnicity				0.469
HL	6 (3.80%)	5 (4.20%)	1 (2.56%)	
NHL	142 (89.87%)	108 (90.76%)	34 (87.18%)	
Unknown/declined	10 (6.33%)	6 (5.04%)	4 (10.26%)	
Charlson Comorbidity Score (median, IQR)	2.00 (0.00, 6.00)	3.00 (0.00, 6.00)	0.00 (0.00, 2.00)	
STEMI	32 (20.25%)	7 (5.88%)	25 (64.10%)	0.006
Clinical History				
Congestive Heart Failure	52 (32.91%)	48 (40.34%)	4 (10.26%)	<0.001
Diabetes	54 (34.18%)	46 (38.66%)	8 (20.51%)	0.038
Cerebrovascular Accident	16 (10.13%)	13 (10.92%)	3 (7.69%)	0.809
CKD	34 (21.52%)	32 (26.89%)	2 (5.13%)	0.004
Prior MI	9 (5.70%)	8 (6.72%)	1 (2.56%)	0.330
Hypercholesterolemia	18 (11.39%)	16 (13.45%)	2 (5.13%)	0.156
Hyperlipidemia	19 (12.03%)	14 (11.76%)	5 (12.82%)	0.860
Aortocoronary bypass graft	18 (11.39%)	15 (12.61%)	3 (7.69%)	0.302
Cardiac Defibrillator	7 (4.43%)	6 (5.04%)	1 (2.56%)	0.448
Heart Device	1 (0.63%)	1 (0.84%)		1
Heart Valve Replacement	3 (1.90%)	2 (1.68%)	1 (2.56%)	0.849
Pacemaker	7 (4.43%)	6 (5.04%)	1 (2.56%)	0.448
Vascular Angioplasty Implant graft	7 (4.43%)	4 (3.36%)	3 (7.69%)	0.572
Smoking Status				0.071
Current	20 (12.66%)	14 (11.76%)	6 (15.38%)	
Former	33 (20.89%)	29 (24.37%)	4 (10.26%)	
Lactic Acid	6.69 (4.42)	7.04 (4.70)	5.68 (3.34)	0.249
Lactate	7.69 (4.05)	7.89 (4.15)	6.95 (3.66)	0.290
Serum Creatinine	2.28 (2.10)	2.57 (2.31)	1.38 (0.77)	<0.001
Creatinine POC	2.21 (1.78)	2.44 (1.87)	1.05 (0.21)	0.011
CAHP Score range				<0.001
High (> 200)	61 (38.61%)	56 (47.06%)	5 (12.82%)	
Medium (150-200)	55 (34.81%)	40 (33.61%)	15 (38.46%)	
Low (< 150)	42 (26.58%)	23 (19.33%)	19 (48.72%)	

Clinical outcomes 

In-Hospital Mortality

**Figure 1:**
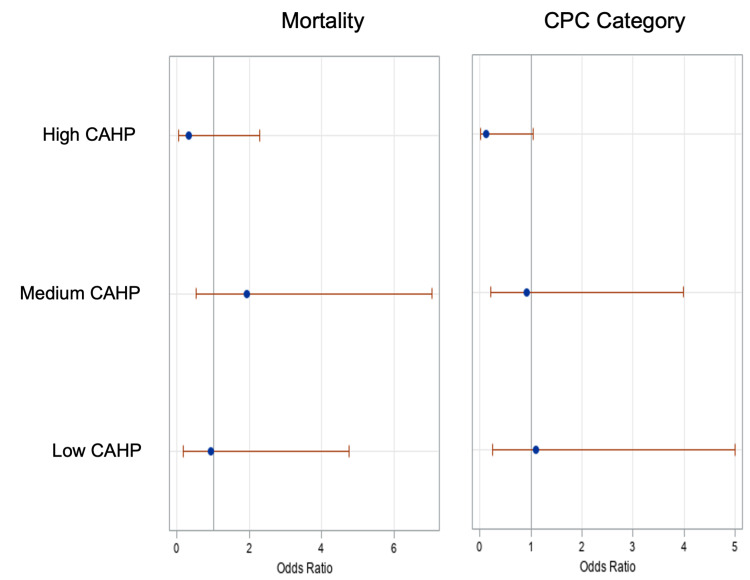
Odds Ratio Estimate of Mortality (left) and Unfavorable Cerebral Outcome (right) for Early vs Delayed Catheterization Conditioned on Low, Medium and High CAHP Scores

Clinical outcomes at the time of hospital discharge are summarized in Tables 2-4. The overall unadjusted in-hospital mortality in the cohort was 56%. Patients who underwent early catheterization were less likely to die than those who underwent delayed or no cardiac catheterization (41% vs 61% mortality, p=0.02). However, the mortality differences were not significant within the subgroup of patients with low, medium, and high CAHP scores (Table 3). The overall unadjusted in-hospital mortality in patients who underwent early catheterization vs delayed or no catheterization was 15.8% vs 17.4% (p=0.88), 66.7% vs 55 % (p=0.39), and 60.0 % vs 83.9% (p=0.212) in subgroup of patients with low, medium, and high CAHP scores. In the multivariable logistic regression model adjusted for age, sex, Charlson comorbidity scores, and CAHP scores, the odds ratio of mortality for early vs delayed/no catheterization were 1.15 (CI: 0.45-2.96), p=0.82 in the overall population. The interaction term of catheterization time and CAHP score was not significant (p = 0.278) when introduced in the multivariable model that adjusts for age, sex, Charlson comorbidity scores, and CAHP scores (Figure 1). 

**Table 2:** Comparisons of Outcomes Between Early and Delayed/Not Catheterized Patients

Variable	All (n=158)	Delayed/No Catheterization (n=119)	Early Catheterization (n=39)	P-value
Mortality, n %	89 (56.33%)	73 (61.34%)	16 (41.03%)	0.026
Cerebral Performance Scale				<0.001
Favorable	50 (31.65%)	29 (24.37%)	21 (53.85%)	
Unfavorable	108 (68.35%)	90 (75.63%)	18 (46.15%)	
Risk of poor outcome	0.76 (0.28)	0.81 (0.24)	0.59 (0.31)	<0.001
Length of Stay	5.75 (1.29, 10.88)	5.04 (0.79, 10.04)	6.50 (2.71, 12.71)	<0.136

**Table 3:** Un-Adjusted Mortality Comparison Between Early and Delayed Catheterization Conditioned on CAHP Scores

CAHP score range	Catheterization Timing	Mortality		Mortality percentage	p-value
		Survived	Died		
Low (< 150)	Delay	19	4	17.4	0.889
	Early	16	3	15.8	
Medium (150-200)	Delay	18	22	55.0	0.393
	Early	5	10	66.7	
High (>200)	Delay	9	47	83.9	0.212
	Early	2	3	60.0	

**Table 4:** Un-Adjusted Cerebral Performance Category Score Outcome Comparison Between Early and Delayed Catheterization Conditioned on CAHP Scores

CAHP score range	Catheterization Timing	Cerebral Outcome		Unfavorable (%)	p-value
		Favorable	Unfavorable		
Low (<150)	Delay	18	5	21.74	0.957
	Early	15	4	21.05	
Medium (150-200)	Delay	8	32	80.00	0.772
	Early	4	11	73.33	
High (>200)	Delay	3	53	94.64	0.049
	Early	2	3	60.00	

Cerebral Performance Scores on Discharge

The final pre-hospital discharge CPC score for the entire cohort was favorable in 31.6% of patients and unfavorable in 68.3% of patients. Patients who underwent early catheterization were less likely to have unfavorable CPC at hospital discharge compared to the patients with delayed or no catheterization (46.1% vs 75.6%, p < 0.001). The unadjusted unfavorable cerebral performance score at the time of discharge among the patients who underwent early catheterization vs delayed catheterization was 21.1 % vs 21.7% (p = 0.95), 73.3% vs 80.0 (p = 0.77) in a subgroup of patients with low, and medium scores respectively indicating no benefit with early cardiac catheterization. However, in the high CAHP score subgroup, there were higher number of patients in the delayed/no catheterization group (60 vs. 94.6%, p = 0.04) who had an unfavorable CPC score on discharge indicating a potential benefit of early catheterization in this patient population (Table 4). However, in the multivariable logistic regression risk-adjustment model (adjusted for age, gender, Charlson comorbidity scores, catheterization timing, and CAHP scores), the odds ratio 0.69 (CI: 0.25-1.89, p=0.477) of unfavorable cerebral outcomes for early vs delayed/no catheterization was not suggestive of any benefit in the overall population. The interaction effect of the CAHP score range and catheterization timing was not significant when introduced in the model. Thus, when adjusted for baseline risk, there is no benefit of early catheterization on cerebral performance category on discharge in the overall population, and that this effect is consistent across the patient’s population with different CAHP score ranges.

CAHP Score Performance

**Figure 2:**
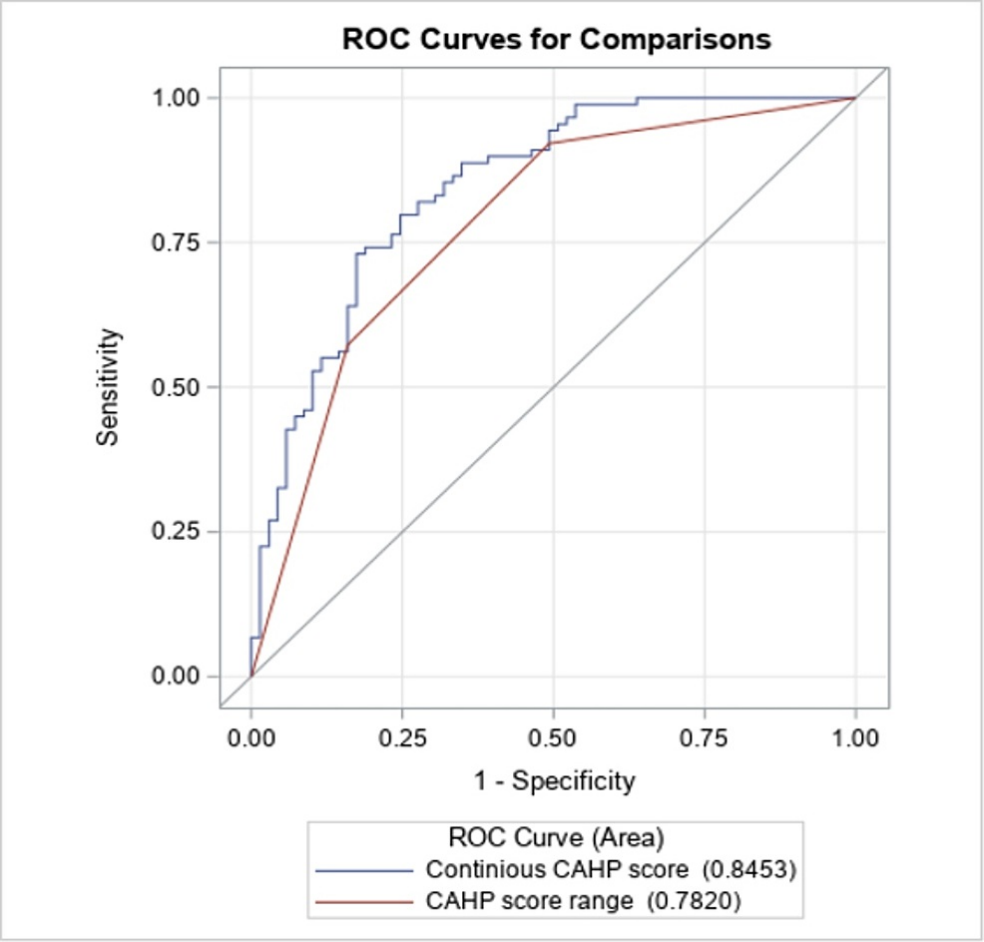
CAHP Score Validation for Mortality The CAHP score (in continuous scale) predicts mortality with an accuracy of 84.5% (C-statistic 0.845) and the CAHP score range (grouped in to high, medium and low level) predict the mortality with an accuracy of 78.20% (C-statistic 0.782).

ROC analysis using this dataset found the CAHP score as a continuous variable predicted mortality with excellent discrimination (c-statistic was 0.85) (Figure 2). Discrimination was also excellent when using CAHP score ranges (low, medium, and high; c-statistic 0.78). The CAHP score as a continuous scale predicted a favorable vs unfavorable cerebral performance scale outcome with an accuracy of 90.5% and the CAHP score range (low, medium, or high) predicted the outcome with an accuracy of 83.2%.

## Discussion

The most important finding of our study is that in a consecutive series of OHCA patients stratified by CAHP prognostic scoring system, there was no difference in risk-adjusted mortality between patients who underwent early cardiac catheterization and those who did not. Use of early cardiac catheterization was associated with a greater likelihood of favorable neurologic recovery in patients with high CAHP scores, but the difference became statistically insignificant when risk-adjustment for selected baseline characteristics (of age, gender, Charlson comorbidity score, catheterization timing, and CAHP score group). 

Among comatose OHCA patients with ROSC, there are no randomized clinical trials (RCT) that demonstrate favorable neurological outcomes or survival benefits with immediate angiography [4]. Even in patients who have underlying ischemic heart disease-causing OHCA, a substantial portion may have chronic ischemic heart disease without new acute coronary occlusion. This subset of patients may have severe coronary artery disease on angiography, but it is not clear whether and when percutaneous coronary intervention (PCI) may improve outcomes [6,7]. Studies suggest that a decision about whether to offer early coronary angiography after OHCA should be based on an overall estimate of survival as assessed by a prognostic scoring system [8]. A recent systematic review by Verma et al. (2020), revealed that early coronary angiography did not impact mortality or neurological status in patients with out-of-hospital cardiac arrest [9].

ECG evidence of STEMI may influence practitioners to pursue early cardiac catheterization since this condition can be readily reversed with invasive therapies. In our study population, more patients with STEMI underwent early coronary angiography. It is possible that this STEMI population influenced the overall benefit of unadjusted mortality in early coronary angiography patients. It is unlikely that a randomized trial involving OHCA patients with STEMI to define the impact of early angiography on outcomes in different CAHP score groups can be done ethically. Therefore, observational studies must be used to inform clinical practice. 

In this retrospective study, we observed clinical practices that were largely consistent with care recommendations expressed in a recent expert consensus statement for patients with OHCA [4]. When deciding whether to offer invasive treatments, it is prudent to consider the presence of co-morbidities that portend unfavorable short- and long-term prognoses, including advanced age, severe dementia, chronic advanced respiratory failure, severe frailty or disability, end-stage renal or liver disease, and advanced metastatic malignancy [4]. While a formal prognostic scoring system was not in place at our institution between 2016 and 2019, these observations suggest that clinicians were intuitively using the elements of the CAHP score when considering the use and timing of cardiac catheterization, since patients with higher CAHP scores were less likely to undergo early angiography.

To our knowledge, a formal evaluation of the CAHP scoring system, which was developed in France, has not been performed previously in a US patient population. Our study indicates that the CAHP score is reliable when used as either a continuous scale or a categorical range variable in predicting mortality with a high degree of accuracy. We, therefore, feel that there is a significant benefit of utilizing this scoring system for OHCA patients on arrival to the hospital to guide cardiac catheterization strategy as well as future research studies.

Limitations

There are several important limitations to this study. This is a retrospective observational study, subject to the limitations of all such studies. Unmeasured factors may be present and influential in clinical decision-making regarding the use of invasive cardiac services. A majority of the potential subjects were not included in the analysis because of insufficient documentation of elements required to calculate the CAHP score. We did not have direct access to documentation from pre-hospital emergency personnel, unlike care systems in France and elsewhere. The majority of the patients in this study were white and male; caution should be used when extrapolating findings to other groups. The study is small, and our ability to detect meaningful differences in outcomes was limited. Because of these limitations, the associations we observed may not indicate causality; rather, these observations should be considered hypothesis-generating. Additional observational reports from other sources are needed to affirm or refute our findings.

## Conclusions

We observed that when adjusted for baseline risk factors, early use of cardiac catheterization in survivors of OHCA did not result in a meaningful mortality benefit or neurologic recovery in any of the CAHP score based sub-groups. Given ethical concerns about conducting randomized trials in OHCA patients with STEMI and high CAHP scores, further observational studies with good capture of cardiopulmonary resuscitation procedure data and outcomes after coronary angiography are needed critically. When patients were arranged into mortality risk categories as predicted by the CAHP scoring system, survival was greatest for those with low-risk scores and lowest for those with high-risk scores. We demonstrated a good fit between observed outcomes and outcomes predicted by the CAHP scoring system, suggesting this simple scoring system may have utility in clinical decision making for this difficult patient group. 
